# Thalidomide combined with low‐dose short‐term glucocorticoid in the treatment of critical Coronavirus Disease 2019

**DOI:** 10.1002/ctm2.35

**Published:** 2020-06-04

**Authors:** Chengshui Chen, Feng Qi, Keqing Shi, Yuping Li, Ji Li, Yongping Chen, Jingye Pan, Tieli Zhou, Xiangyang Lin, Jinsan Zhang, Yongde Luo, Xiaokun Li, Jinglin Xia

**Affiliations:** ^1^ COVID‐19 Headquarters The First Affiliated Hospital of Wenzhou Medical University Wenzhou China; ^2^ Liver Cancer Institute, Zhongshan Hospital Fudan University Shanghai China; ^3^ International Collaborative Center on Growth Factor Research Wenzhou Medical University Wenzhou China; ^4^ School of Pharmaceutical Sciences Wenzhou Medical University Wenzhou China

**Abbreviations**
ARDS: acute respiratory distress syndrome
IFN: interferon
IL: interleukin
MERS: Middle East respiratory syndrome
rRT‐PCR: real‐time reverse‐transcriptase polymerase chain reaction
COVID‐19: coronavirus disease 2019
SARS‐CoV‐2: severe acute respiratory syndrome coronavirus 2

An epidemic illness caused by severe acute respiratory syndrome coronavirus 2 (SARS‐CoV‐2), now named Coronavirus Disease 2019 (COVID‐19), occurred in Wuhan, China.^1^ The human‐to‐human contagious transmission of SARS‐CoV‐2 has been confirmed, leading to rapid spreading to tens of thousands of patients in China and other regions in the world.^2^, ^3^, ^4^, ^5^ Organ dysfunction, including acute respiratory distress syndrome (ARDS), shock, and death may occur.^6^ Therefore, many COVID‐19 patients also suffered from anxiety, especially under treatment in intensive care units. However, due to currently very limited treatment options and no developed vaccines available for COVID‐19, new treatment approaches are urgently needed. There were quite a few critical cases suffering from immune imbalance,^7^ for which the efficacy of antiviral drugs might remain unsatisfactory or insufficient, especially in the later stages of disease progression. Thalidomide, referred to phocomelia, has been introduced as an anti‐inflammatory therapy with remarkable efficacy in many autoimmune disorders, such as psoriasis, systemic lupus erythematosus, and inflammatory bowel disease, in which the suppressive effect of thalidomide on the pro‐inflammatory cytokines, including interleukin (IL)‐6, tumor necrosis factor (TNF)‐α, and interferon (IFN), was revealed.^8^, ^9^, ^10^ In addition, thalidomide has been known for its co‐stimulatory effect on proliferation of T cells following CD3 activation.^11^ Based on the effect of reducing pro‐inflammatory cytokines and maintaining immune homeostasis of thalidomide, we introduced this drug for treatment of the patients with critical/severe COVID‐19 pneumonia for whom the efficacy of antiviral drugs might remain unsatisfactory or insufficient, especially in the late stage. Here, we report the protective effect of thalidomide in combination with antiviral drugs and low‐dose short‐term glucocorticoid on lung injury and immunological dysfunction caused by critical COVID‐19.

On 31 January 2020, a 45‐year‐old woman was admitted to a fever clinic of Wencheng County People's Hospital, in Wenzhou city, Zhejiang province, with a 5‐day history of cough, fever, fatigue, and diarrhea. She denied any recent travel to Wuhan, China, or close contact with infected persons or suspected cases. The patient exhibited no dyspnea. She was first treated with ofloxacin and oseltamivir, but the condition deteriorated. The swab specimen was tested positive for SARS‐CoV‐2 by real‐time reverse‐transcriptase polymerase chain reaction (rRT‐PCR) on 1 February 2020. Chest computerized tomography indicated signs of the subpleural effusions in the left upper and left lower lung (Figures 1A and 1B). Therefore, the patient was diagnosed with COVID‐19, and treated with lopinavir/ritonavir. Due to the persistent hyperpyrexia, she was transferred to the isolation ward in our hospital on 3 February 2020 for further treatment. The patient was healthy before this outbreak. Physical examination revealed a body temperature of 38.1°C, blood pressure of 117/78 mmHg, pulse rate 92 beats per minute, and a respiratory rate of 20 breaths per minute. On admission, the patient's vital signs were initially stable. This patient continued to have a high fever, dyspnea, and was obviously fatigued, accompanied by nausea and vomiting. Treatment during this period was primarily supportive and antiviral therapy. However, on hospital day 2 (illness day 6), oxygen saturation decreased to 93% while the patient was treated by nasal cannula delivery of oxygen at 3 L/min, and arterial blood gas analysis indicated a deterioration of the oxygenation index (PaO_2_/FiO_2_: 220 mmHg). According to Novel Coronavirus Infection Pneumonia Diagnosis and Treatment Standards (the sixth edition), the patient was classified into the critical phenotype.

**FIGURE 1:**
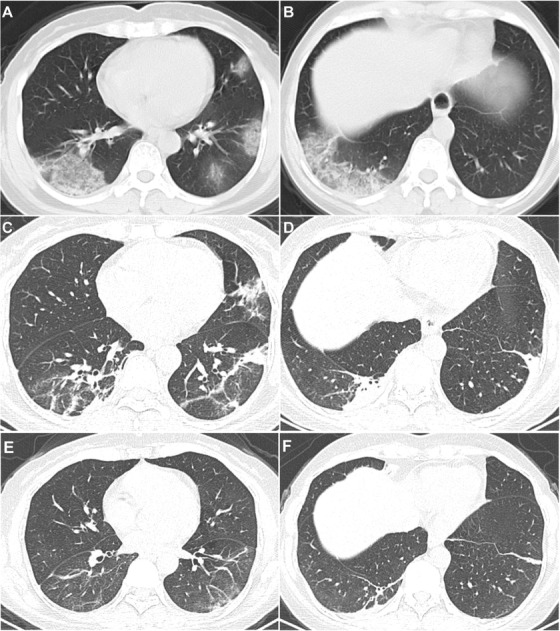
Chest computed tomography images. A and B, Subpleural exudation opacities in the lower right, left upper lung and left lower lung, on 2 February 2020. C and D, Fibrous lesions in the lower right, left upper lung, and left lower lung, on 11 February 2020. E and F, Fibrous lesions in the lower right, left upper lung and left lower lung, on 17 February 2020

Laboratory testing revealed a significantly increased level of C‐reactive protein at 90.0 mg/L and cytokine levels including IL‐6 at 102.95 pg/mL, IL‐10 at 24.84 pg/mL, and IFN‐γ at 38.16 pg/mL (Figure 2A). Lymphocytopenia appeared, as well as a significantly decreased T cell absolute value (254/T cell µL), including CD4+ T cells (163/µL), CD8+ T cells (83 /µL), NK cells (44 /µL), and B cells (76 /µL) (Figure 2B). These results indicated that cytokine surge and inappropriate immune response occurred in this patient.

**Headlights**

- Host immune responses are important factors leading to life‐threatening organ dysfunction in patients with Coronavirus Disease 2019.
- The pulmonary effusion and elevated serum inflammatory cytokines were substantially reduced and lymphocytes recovered by the treatment of thalidomide combined with a low‐dose short‐term glucocorticoid.
- Thalidomide may be a new dawn as an adjuvant treatment strategy for Coronavirus Disease 2019.

**FIGURE 2:**
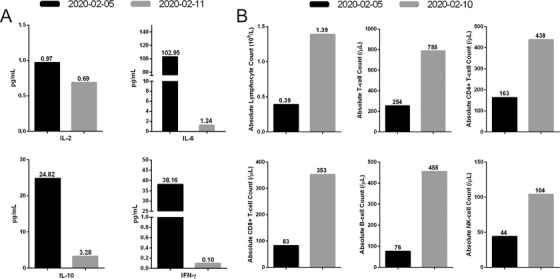
Inflammatory cytokines and lymphocytes in serum before and after thalidomide treatment. A, Inflammatory cytokines: The normal ranges are as follows: IL‐2 ˂ 3.10 pg/mL; IL‐6 ˂ 3.00 pg/mL; IL‐10 ˂ 4.10 pg/mL; and IFN‐γ ˂ 2.20 pg/m. B, Lymphocytes: The normal ranges are as follows: T‐cell absolute value: 797‐2370/µL; CD4+ T‐cell absolute value: 432‐1341/µL; CD8+ T‐cell absolute value: 238‐1075/µL; B‐cell absolute value: 86‐594/µL; NK‐cell absolute value: 127‐987/µL Abbreviations: IFN, interferon; IL, interleukin.

Because of the exacerbation of the disease and the aggressive immune response, treatment with thalidomide (Changzhou Pharmaceutical Factory Co., Ltd. Jiangsu, China) at 100‐mg dose orally every 24 h and low‐dose short‐term methylprednisolone (40 mg administered intravenously every 12 h for 3 days and then reduced to every 24 h for 5 days) was initiated on 5 February 2020, hospital day 2. No adverse events were observed. After the treatment of this strategy, the cytokine levels returned to the normal range including IL‐6 at 1.24 pg/mL, IL‐10 at 3.28 pg/mL, and IFN‐γ at 0.10 pg/mL on 11 February 2020 (Figure 2A). The lymphocyte absolute value increased from 0.39 × 10^9^/L to 1.39 × 10^9^/L, T cells from 254 to 788/µL, CD4 + T cells from 163 to 438/µL, CD8+ T cells from 83 to 353/µL, NK cells from 44 to 104/µL, and B cells from 76 to 455/µL on 10 February 2020 (Figure 2B). The dynamic course of changes in the total amount of white blood cells and the oxygen index during hospitalization were shown in Figure 3A. Furthermore, the patient's clinical condition improved, including disappearance of anxiety and nausea and vomiting after thalidomide treatment (Figure 3B). The oxygen index rose to 402 mmHg. As of 12 February 2020, the previous exudation of the left lung was decreased significantly (Figures 1C and 1D) and the patient returned to afebrile. The SARS‐CoV‐2 tests in swab specimen on 11 February 2020 and on 14 February 2020 and in feces on 12 February 2020 were negative. The lesions in lung almost disappeared on 17 February 2020 (Figures 1E and 1F), and the patient was discharged. The outbreak of COVID‐19 rapidly spread throughout the globe, which disrupted public healthcare systems worldwide. By 20 April 2020, there were 84 237 patients, 4642 death, and 77 895 cured patients in China, and 2 313 996 patients, 160 504 death, and 545 326 cured patients in countries other than China. Through prevention and control measures, there are only 1700 existing confirmed cases in China. However, due to strong infectivity and epidemic intensity, 1 608 166 existing confirmed cases still remained in other regions out of China. Currently, no therapeutic strategy was approved for treating COVID‐19. Some potential antiviral drugs, such as Remdesivir and Hydroxychloroquine, have been recommended in COVID19. In a compassionate use study, although clinical improvement was observed in 68% patients with severe COVID‐19, 60% patients had adverse events and 23% patients had serious adverse events, including multiple organ dysfunction syndrome, septic shock, and acute kidney injury.^12^ Hydroxychloroquine could improve radiological progression of lung disease; however, the effects of hydroxychloroquine on virological cure and preventing progression to severe disease were not observed.^13^ In addition, this agent can cause rare and serious adverse effects, including QTc prolongation, hypoglycemia, neuropsychiatric effects, and retinopathy.^14^ It has been well documented that host immune responses are important factors leading to life‐threatening ARDS in COVID‐19 patients.^7^, ^15^ Therefore, the adjunctive therapies for regulating immune should be considered. Given the reported anti‐inflammatory and immunomodulatory effects of thalidomide, we sought to treat this patient who had developed a critical COVID‐19 with thalidomide in combination with low‐dose short‐term glucocorticoid. We reported here that this therapeutic strategy had a beneficial outcome in this critical patient.

**FIGURE 3:**
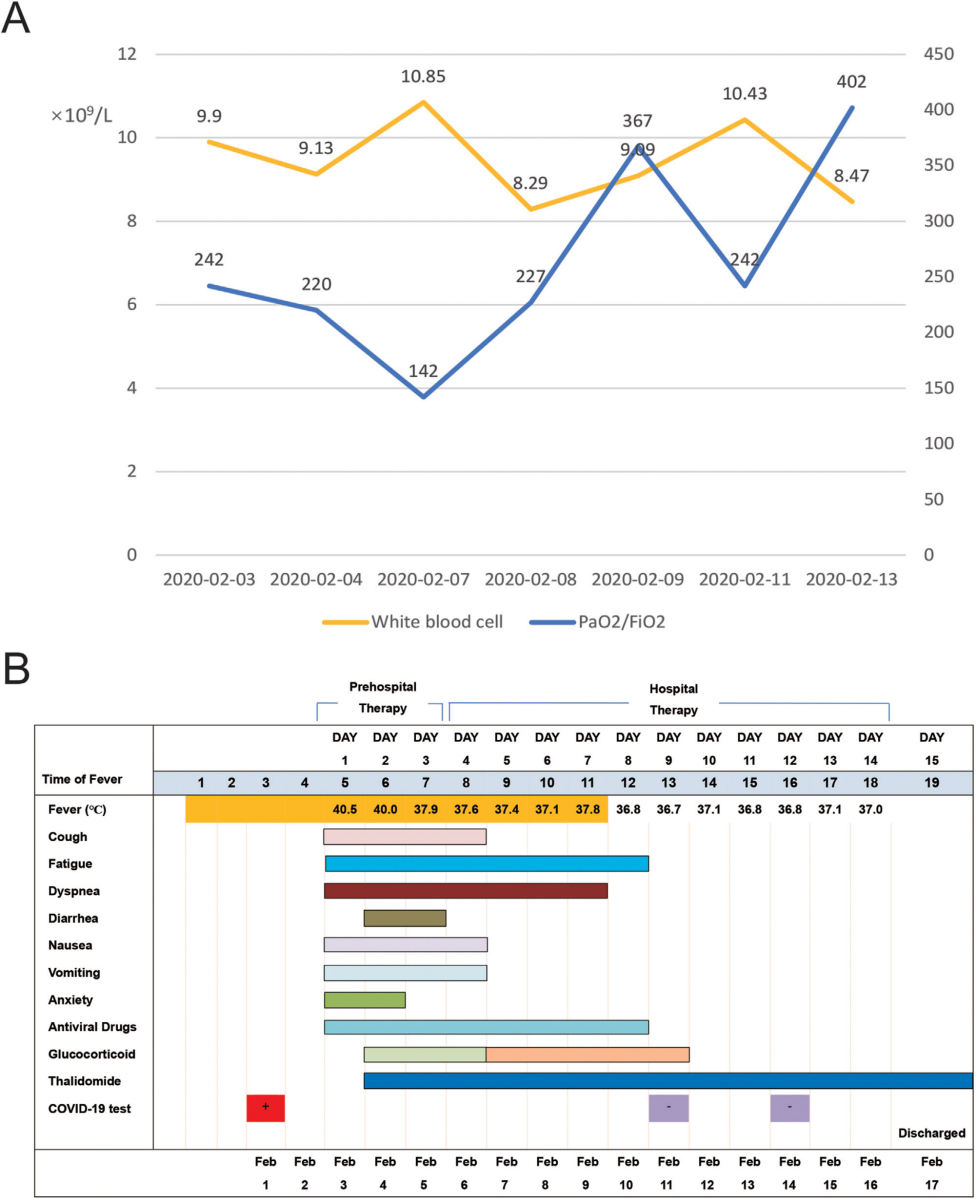
White blood cells, oxygen index, symptoms, body temperatures, and drug use during the treatment. A, Dynamic course of the total number of white blood cells and the oxygen index during hospitalization. B, Symptoms, body temperatures, and drug use according to the day of illness, 3 February to 17 February 2020

In current case, acute pulmonary effusion was observed due to markedly elevated inflammatory cytokine profiles in the serum including IL‐6, IL‐10, and IFN‐γ, manifesting as a cytokine surge that is caused by rapidly proliferating and highly activated T cells and could subsequently lead to tissues damage and organs failure. High dose of Glucocorticoid is usually used for suppressing cytokine surge, which therefore was widely applied during the outbreaks of Severe Acute Respiratory Syndrome (SARS) and Middle East Respiratory Syndrome (MERS) CoV infections to suppress lung inflammation and immune responses.^16^ However, it appeared to be associated with treatment side effects, such as secondary bacterial infection, osteoporosis, and others. Therefore, glucocorticoid was not recommended for critical COVID‐19 as used in SARS‐CoV or MERS‐CoV infections,^17^ also due to its inhibition of immune responses and pathogen clearance. Another potential immune regulator for COVID‐19 is the use of convalescent plasma, which is conducive to virus and infected cell immune clearance.^18^. However, the shortage of convalescent plasma limits its widespread application. Monoclonal antibodies that could directly reduce inflammatory cytokines may theoretically dampen organ damage caused by cytokine release and improve clinical outcomes of COVID‐19. However, the effect and safety of monoclonal antibodies were not evaluated in clinical trials. Interestingly, we found that after combined treatment of thalidomide with low‐dose short‐term glucocorticoid, the pulmonary effusion symptoms and elevated inflammatory cytokines in the present patient were substantially reduced without any side effect. Simultaneously, the number of lymphocytes recovered. These results indicated that thalidomide could be used in conjunction with low‐dose short‐term steroids to treat a critical COVID‐19, likely based on its known anti‐inflammatory and immunoregulatory effects. Previous studies indicated that thalidomide inhibited lung injury in mice with H1N1 influenza virus, improved survival, reduced inflammatory cell infiltration, and inhibited cytokines (IL‐6 and TNF‐α).^19^ It was also shown that thalidomide could activate T cell receptors and T cells to enhance immune functions.^11^ Therefore, thalidomide not only alleviates organ injury by inhibiting the cytokine “storm” but also improves immune functions to reduce secondary bacterial infections. Furthermore, the beneficial effect of thalidomide on COVID‐19 might also be attributable to its sedative and antiemetic activities that help an anxious patient calm down to reduce oxygen consumption, and alleviate digestive symptoms in this patient.

## 1 CONCLUSION

Thalidomide could not only be used to inhibit cytokine surge and regulate immune functions, but also be used to calm patients down to reduce oxygen consumption, and relieve digestive symptoms in COVID‐19 patients. Therefore, thalidomide may be a new dawn as an adjuvant treatment strategy for patients with critical COVID‐19. A randomized controlled trial to investigate the effectiveness of thalidomide combined with low‐dose short‐term glucocorticoid for COVID‐19 treatment needs to be performed.

## CONFLICT OF INTEREST

The authors declare no conflict of interest.

## INFORMED CONSENT

Written informed consent was obtained from the participant included in this study.

## AUTHOR CONTRIBUTIONS

JLX and XKL conceived, designed, and supervised the overall study. CSC, YPL, JL, YPC, and JYP collected clinical data. FQ and KQS analyzed and interpreted the data. JLX, JL, and YPL formulated the treatment regimen and analyzed the computerized tomography images. TLZ and XYL did the flow cytometric analysis. FQ and KQS made the figures and wrote the manuscript. JLX, JSZ, and YDL critically revised the manuscript.
